# Clinical signs of brachycephalic ocular syndrome in 93 dogs

**DOI:** 10.1186/s13620-021-00183-5

**Published:** 2021-01-25

**Authors:** Joana Costa, Andrea Steinmetz, Esmeralda Delgado

**Affiliations:** 1 Faculty of Veterinary Medicine, University of Lisbon, Lisbon, Portugal; 2 Department of Small Animals, Faculty of Veterinary Medicine, University of Leipzig, Leipzig, Germany; 3 CIISA- Centre for Interdisciplinary Research in Animal Health, Faculty of Veterinary Medicine, University of Lisbon, Lisbon, Portugal

**Keywords:** Brachycephalic ocular syndrome; brachycephalic breeds, Medial entropion; corneal pigmentary keratitis, Corneal fibrosis, Corneal ulcers

## Abstract

**Background:**

Brachycephalic breeds have anatomical skull changes that are responsible for ocular clinical signs, known as the brachycephalic ocular syndrome (BOS). Their popularity has increased in recent years but the excessive pressure of selection lead to extreme conformation of skull shapes, resulting in facial alterations that can put these dogs’ vision at risk.

**Objectives:**

This study aimed to analyse the ocular disorders in a sample of 93 brachycephalic dogs to better characterize the disease complex BOS.

**Material and methods:**

Brachycephalic dogs were submitted to a complete ophthalmological examination. The studied parameters included animal’s sex, age and breed, age, ophthalmological tests performed, results of complementary exams, clinical signs, ocular disorders, treatment protocols and their outcomes. Data were organized using Microsoft Office Excel 2007® and statistical analysis was performed with IBM SPSS Statistics 20®.

**Results:**

The studied population included 93 brachycephalic dogs 45 males (48%) and 48 females (52%) from different breeds: French Bulldog (*n* = 38), Shih-Tzu (*n* = 22), Pug (*n* = 17), English Bulldog (*n* = 5), Pekingese (*n* = 4), Boxer (n = 4) and Boston Terrier (*n* = 3), aged between 0.2–16 years, median 4.65 years. The most frequent ocular abnormalities were corneal ulcers in 44%, corneal pigmentation in 36%, corneal fibrosis in 25% and entropion in 22% of the animals. There was a higher incidence of corneal pigmentary keratitis in Pugs (53%) and corneal fibrosis in Shih Tzus (36%). The most common surgical techniques were medial canthoplasty in 22%, conjunctival flap in 10% and electroepilation in 7% of the cases, without post-operative complications. Conclusions: This study contributed to a better characterization of the disease complex brachycephalic ocular syndrome. The percentage of ocular disorders like entropion, corneal pigmentation, fibrosis and ulcers was high, highlighting the importance of a regular ophthalmological check-up, and early diagnosis of the primary disorders. A higher incidence of corneal pigmentation was noticed in Pugs and corneal fibrosis in Shih Tzus, which suggests that some brachycephalic breeds may be predisposed to certain ocular abnormalities. A responsible reproductive strategy should be implemented to avoid undesired transmission of the abnormal traits to the offspring.

## Introduction

The brachycephalic breeds are spread around the world and their popularity has increased in recent years. The scientific word used to describe short-nosed or flat-faced dogs originated from two Greek words meaning ‘short’ and ‘head’. The number of these patients is increasing in small animal practices. Their personalities, wrinkly faces and appealing large eyes have turned them into popular pets. This popularity is thought to exist because humans find the large and round eyes, as well as the round face very appealing [1].

Pedigree dogs are artificially selected for aesthetics dictated by formal breed standards, and breed-related disorders as a result are diverse. The pressure of selection applied specially over the last three decades led to the development of dramatic changes to the brachycephalic breeds’ skull shapes [2]. The foreshortening of the facial skeleton represents one of those changes and is a discrete mutation that has been selected in many popular flat-faced dogs [3]. This has resulted in several health and welfare problems [4]. Some of these problems include Brachycephalic Airway Obstructive Syndrome and Brachycephalic Ocular Syndrome (BOS) [5–7]; the latter of which is the result of diverse facial alterations, which can originate ophthalmic complications that can put these dogs’ vision at risk [8].

The typical facial and orbital appearance of brachycephalic dogs is characterized by round skull shapes and plane orbits, leading to a physiological degree of exophthalmia. These prominent eyes along with macroblepharon, or excessive long palpebral fissures, don’t allow for an adequate ocular coverage and lubrication. They may frequently present with lagophthalmus, a condition in which the eyelids can’t even close completely, compromising the normal lubrication and protection of the ocular surface. These traits along with decreased corneal sensitivity make the eyes more prone to chronic exposure, resulting in a wide variety of problems, such as trauma, exposure keratopathy, superficial pigmentary keratitis, corneal erosion or even ulceration.

Other common ophthalmic common findings in BOS include entropion, keratoconjunctivitis sicca and eyelash *disorders* such as distichiasis, trichiasis, and ectopic cilia. However, it is important to note that these changes can be found in other breeds, they are not specific to BOS. When not treated, they lead to permanent damage which consequently can cause a decrease of the dogs’ visual acuity and compromise their wellbeing. Some components of the BOS have a genetic background and most of them show their first clinical findings in young to middle-aged dogs, after they become sexually mature. Thus, an undesired transmission of the abnormal trait to their offspring may happen [9].

Known or suspected inherited ocular problems include trichiasis, caruncular trichiasis, distichiasis, ectopic cilia, entropion, excessive nasal fold, decreased corneal sensitivity, corneal dystrophies, prolapse of the gland of the nictitating membrane, keratoconjunctivitis sicca (KCS), hereditary cataracts and progressive retinal atrophy [10]. Again, these changes are not BOS specific.

This prospective study aims to describe a sample of brachycephalic dogs on what concerns their breed, age, gender, clinical signs and ocular alterations, as well as medical and surgical treatment and their outcomes, to better characterize the disease complex ocular brachycephalic syndrome. We intend to highlight the importance of a regular ophthalmological check-up in these breeds, so that an early diagnosis of the primary disorders can be achieved. Also, we want to stress out the need for a responsible reproductive strategy to avoid undesired transmission of the abnormal traits to the offspring.

## Methods

### Study population

Data from medical records of dogs diagnosed with BOS that presented to an ophthalmological consultation at the Ophthalmology Services from the Teaching Hospital of two veterinary medicine schools (University of Lisbon, Portugal and University of Leipzig, Germany) between January 2018 and July 2019, was registered and analysed. Owners gave written consent for inclusion of their animal’s data in this study.

### Ophthalmological examinations

A complete ophthalmic examination was carried out in each patient. The exam included menace response, pupillary, corneal and palpebral reflexes, Schirmer’s Tear Test (Schirmer Tear Test Strips; Eickmeyer, Surrey, United Kingdom), rebound tonometry (Icare Tonovet; Icare Finland, Helsinki, Finland), slit-lamp biomicroscopy (Kowa SL15, Portable Slit-Lamp; Kowa Company, Tokyo, Japan) and direct modified ophthalmoscopy (PanOptic Ophthalmoscope; Welch Allyn, New York, United States of America). When pupillary dilation was necessary, topical tropicamide solution (Tropicil Top 1%; Edol, Linda-a-Velha, Portugal) was used. Moreover, complementary exams were performed in some individuals, such as fluorescein staining (Fluorescein; Haag-Streit International, Köniz, Switzerland), electroretinography and ocular ultrasound.

### Statistical analysis

The studied parameters included animal’s sex, age and breed, age, the results of the ophthalmological tests, complementary exams, the list of all ocular disorders, treatment protocols and their outcomes. Ocular disorders were categorised according to involvement of the eyelids, the cornea and the intraocular structures. Originated data was organized in a database using Microsoft Office Excel 2007® and its statistical analysis was performed using descriptive statistics with the software IBM SPSS Statistics 20®.

## Results

### Study population

The animal population consisted of 93 dogs, including 45 males (48%) and 48 females (52%) with an age range of 0.2–16 years (median 4.65 years). All the dogs were pure-breeds, representing seven different breeds: French Bulldog (*n* = 38), Shih-Tzu (*n* = 22), Pug (*n* = 17), English Bulldog (*n* = 5), Boxer (*n* = 4), Pekingese (n = 4) and Boston Terrier (n = 3). No statistically significant correlation (*p* > 0.05) between the distribution of breed, sex or age and the prevalence of ocular disorders was found.

### Ophthalmological examination and complementary exams

In relation to Schirmer tear test (STT) values, sixteen of the animals had values below the normal range. From these, ten of them presented with values between 10 and 14 mmHg, considered low, and six of them corresponded to severe cases of keratoconjunctivitis sicca (KCS), with STT values varying between 0 and 10 mm/min. This exam, in addition to the remaining clinical signs, allowed for the diagnosis of KCS in a total of eleven animals (11/93).

Tonometry values were also altered in some cases. Twelve of the patients (12/93) presented values outside the reference range, from which six cases were below and six were above it. The low values varied between 5 and 12 mmHg, compatible with uveitis, and the increased ones varied between 40 and 80 mmHg, compatible with glaucoma.

The fluorescein test was the most commonly performed complementary exam, used in eighteen cases of suspected corneal ulcers (18/93).

Ocular ultrasound was used five times (5/93) and allowed for confirmation of two lens changes, one lens luxation and one modification in the vitreous chamber.

Electroretinography was performed in four cases of bilateral cataracts (4/93), and the result was always within the reference range.

Measurement of the arterial pressure was one of the non-ophthalmic complementary exams performed and was crucial to obtain a diagnosis in one dog (1/93), allowing for the confirmation of arterial hypertension, as the value for systolic arterial pressure was 250 mmHg.

#### Ocular disorders

##### Eyelids

Macroblepharon was very common among the population (44/93), affecting almost half of the population; two French Bulldogs presented with extreme phenotypes.

A significant number of patients was affected with eyelid disorders including entropion, excessive nasal folds, ectopic cilia, trichiasis and distichiasis. Among these, entropion was the most frequent problem, with an incidence of 22% (20/93). Not only that, but it affected every breed included in this study, excluding Boxer and Pekingese, and was especially recurrent in Pugs. which represented almost half of the affected dogs, probably due to the fact that it was one of the most well represented breeds in this study. The entropion’s location was always medial and bilateral. In two cases, a Bullmastiffand an English Bulldog, entropion affected not only the inferior eyelid, but also the upper one.

Trichiasis was the second most common eyelid related disorder, being present in fifteen dogs (15/93), from which eleven corresponded to the caruncular type.

**Fig. 1:**
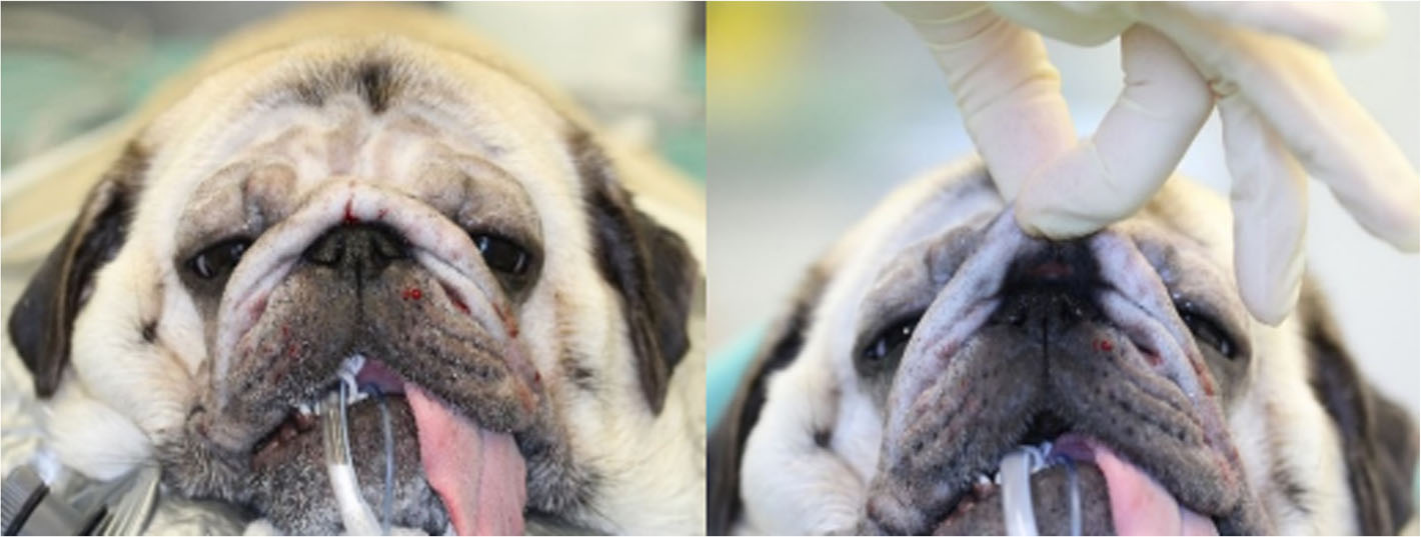
Female Pug with excessive nasal folds. Notice its proximity to the medial canthus

Distichiasis presented an incidence of 16% (15/93), excessive nasal folds were seen in three Pugs and one Pekingese, affecting 4% of the population (4/93) (Fig. 1), and ectopic cilia were only found in one Boston Terrier and one Shih Tzu, concerning 2% of the population (2/93).

Six cases of palpebral masses were registered (6/93), corresponding to one Shih Tzu and five French Bulldogs. Prolapse of the gland of the nictitating membrane was seen in five dogs, unilaterally in one Boston Terrier and in one English Bulldog, and bilaterally in three French Bulldogs.

##### Cornea

**Fig. 2:**
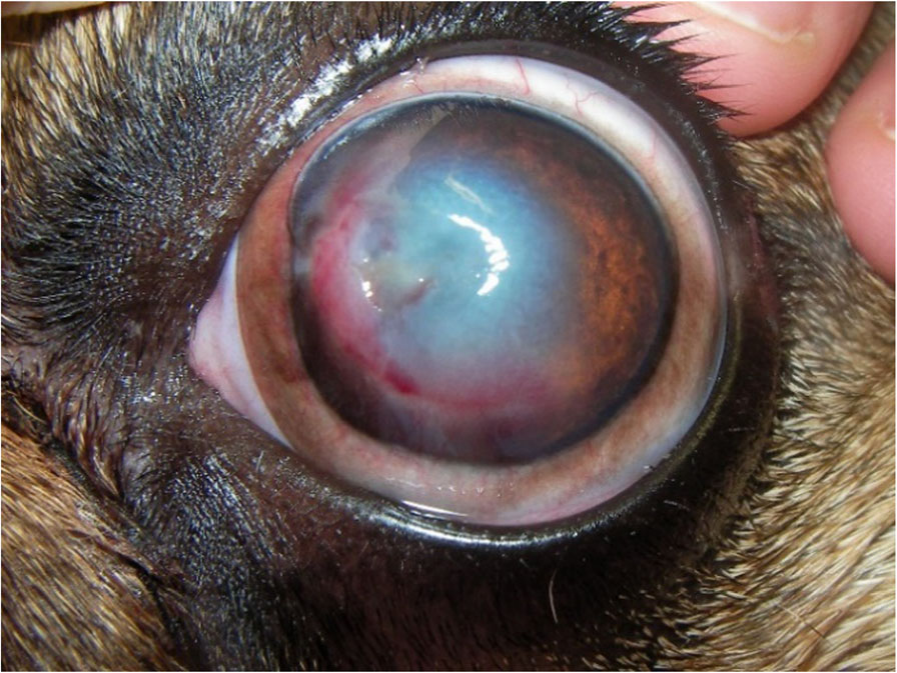
A 2 years old male French Bulldog with a right eye corneal ulcer affecting the mid stroma, accompanied by corneal oedema, neovascularization and granulation tissue

Corneal lesions were quite commonly present and included corneal ulcers, degeneration, pigmentation and fibrosis. Corneal ulcers (41/93) affected the majority of breeds included in the study except for Boston Terrier and Boxer (Fig. 2). From the total of 41 cases, 36 were unilateral and 5 were bilateral. Seventeen of them corresponded to stromal medium or deep lesions, and were diagnosed in seven French Bulldogs and five Shih Tzus, while the remaining corneal ulcers were superficial lesions. Ten cases of eyelash alterations and concomitant corneal ulcers were observed.

Corneal pigmentation (33/93) and fibrosis (23/93) were diagnosed in every breed included in the study, excluding Pekingese.

**Fig. 3:**
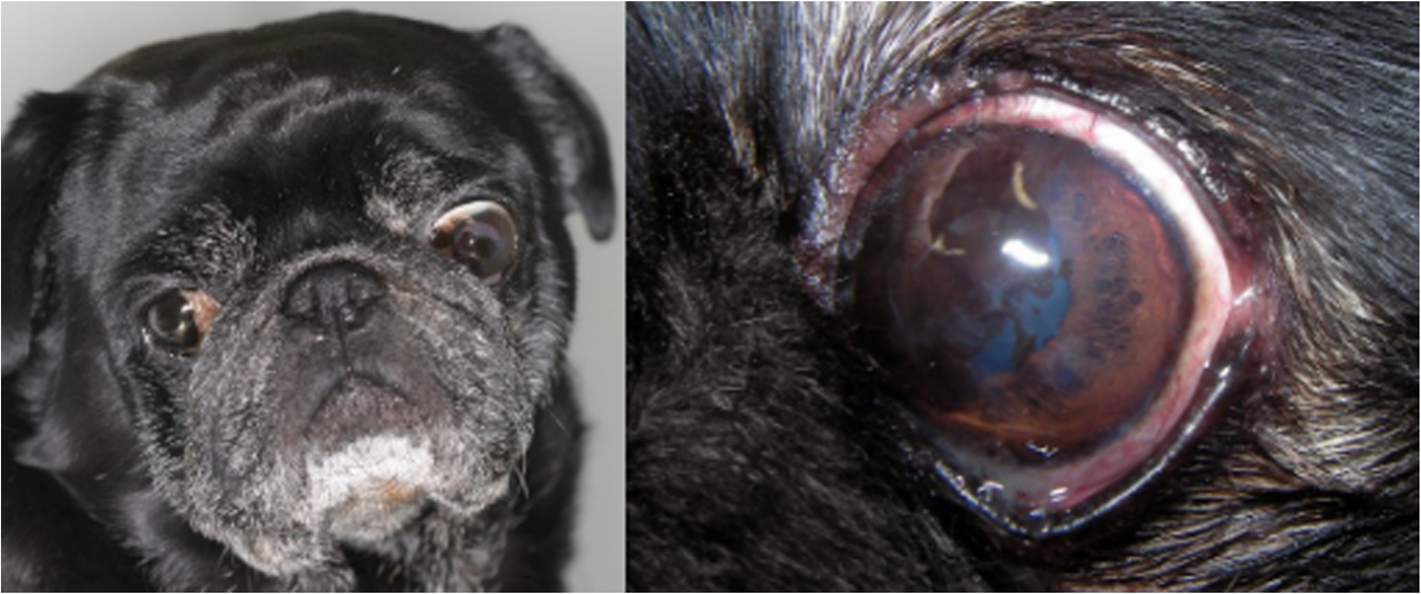
A male Pug with bilateral medial corneal pigmentation. In the right we can see an image of the left eye, showing the superficial pigmentary keratitis affecting the upper and lower medial quadrants of the cornea, and already impairing vision

Corneal pigmentation presented an incidence of 35%, being present in six of the brachycephalic breeds included in the study, and the majority of cases were attributed to Pugs (9/93) and Shih Tzus (8/93) (Fig. 3). The only unaffected breed was the Boston Terrier. The percentage of affected Pugs was 53% and that of Shih Tzus was 36%.

On the other hand, corneal fibrosis had an incidence of 25% and affected mainly Shih Tzus (7/93) and French Bulldogs (10/93). The total percentage of affected Shih Tzus was 36%, and of French Bulldogs was 29%.

Corneal dystrophy was the corneal event that presented with the lowest frequency in this study (6/93).

##### Intraocular disorders

The iris also presented with a low number of lesions, since only five cases of alterations in this ocular structure (5/93) were registered, including Pugs with a sand type pattern in the iris and iris’ discoria, and one Boston Terrier plus one French Bulldog with iris’ cysts.

Two French Bulldogs and two Pekingese dogs presented with lens subluxation and inflammation (4/93), and two Shih Tzus had nuclear sclerosis (2/93), their age being between 10.6 and 16 years old. Anterior chamber abnormalities (3/93) included hyphema and hypopyon. There were also three cases of cataracts seen in three French Bulldogs and two Pugs (5/93) with an age range between of 2 and 10 years old.

#### Treatment protocols

According to clinical presentation, patients were submitted for medical or surgical treatment.

##### Medical treatment

Medical treatment was often indicated, mostly in cases of corneal ulcers, management of KCS and during surgical recovery. Superficial corneal ulcers were systematically treated with topical drugs that involved a combination of lubricating eye drops with polyacrylic acid (Vidisic®- Bausch and Lomb, New York, USA) or hyaluronic acid (Vislube®- TRB Chemedica, Genève, Switzerland) 3-6x/day, topical antibiotic, such as tobramycin (Tobrex®- Alcon, Barcelona, Spain) or gentamicin (Gentamycin-POS®- Ursapharm, Saarbrücken, Germany) 5x/day and atropine (Atropocil®- Edol, Linda-a-Velha, Portugal; Atropin-POS 0,5%®; Ursapharm, Saarbrücken, Germany) SID, with the concomitant use of an Elizabethan collar.

Dogs with deep and severe ulcers were medicated with a topical antibiotic, such as ofloxacin (Oflex®– VAPP, Carnaxide, Portugal; Floxal®, Bausch + Lomb, Berlin, Germany) or chlortetracycline (Cepemycin®- CPPharma, Burgdorf, Germany) 5x/day, lubricating eye drops 3-6x/day, atropine BID and a systemic antibiotic, such as cephalexin for ten days, and then were submitted to a surgical treatment that involved placement of a conjunctival pediculated flap.

If a surgical treatment was not pursued, a conservative medical approach was considered and included the same drugs as in the superficial ulcers, but with a higher frequency of administration, and a placement of a contact lens in the injured eye until healing was achieved.

##### Surgical treatments

**Fig. 4:**
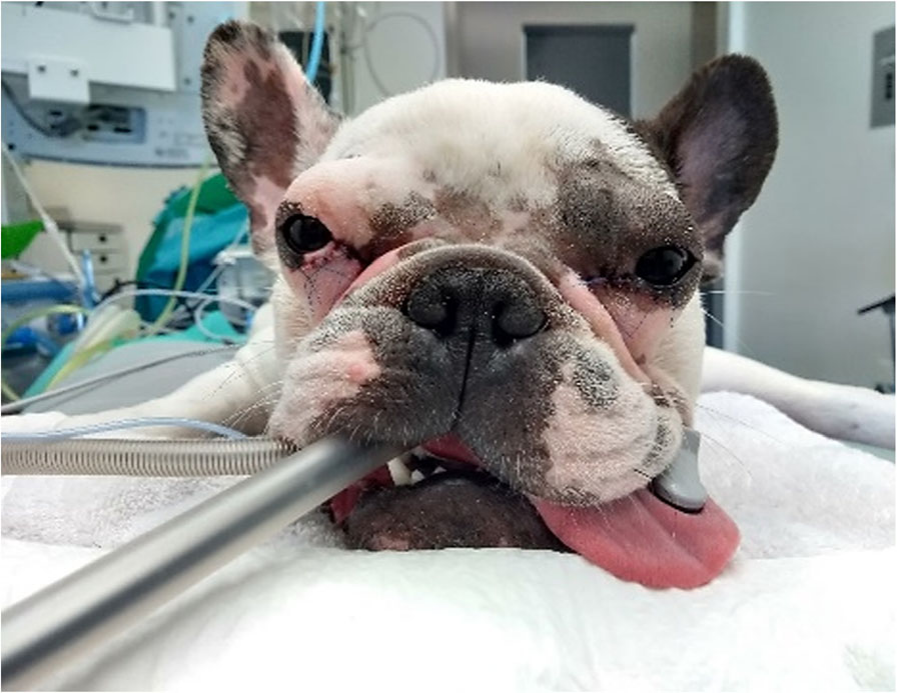
A one-year-old male French Bulldog after surgical correction of the Brachycephalic Ocular Syndrome. A bilateral medial cantoplasty was performed to shorten the eyelid fissure, correcting the lagophthalmia, addressing caruncular trichiasis and correcting the bilateral medial entropion, preserving the lacrimal puncta to avoid epiphora in the future

More than half of the studied population underwentsome type of ophthalmic surgery. The surgical procedure most frequently recommended to address BOS was medial canthoplasty, with twenty indications to do so (22%, 20/93) (Fig. 4). However, surgery was only performed in eleven of the referred cases (55%, 11/20). When considering the surgical approach, it was advised in members from four of the brachycephalic breeds: ten Pugs, six Shih Tzus, three French Bulldogs and one English Bulldog,

Corneo-conjunctival flap was the second most common surgical procedure, being performed nine times (10%, 9/93), from which eight were based on conjunctival tissue and one flap (1%, 1/93) was made from amniotic membrane.

Electroepilation was advised six times (6/93) and manual epilation of distichia and ectopic cilia was needed an additional three times (3/93).

Superficial keratectomy was performed in four cases (4/93) and Hotz-Celsus blepharoplasty was performed in four dogs (4/93).

**Fig. 5:**
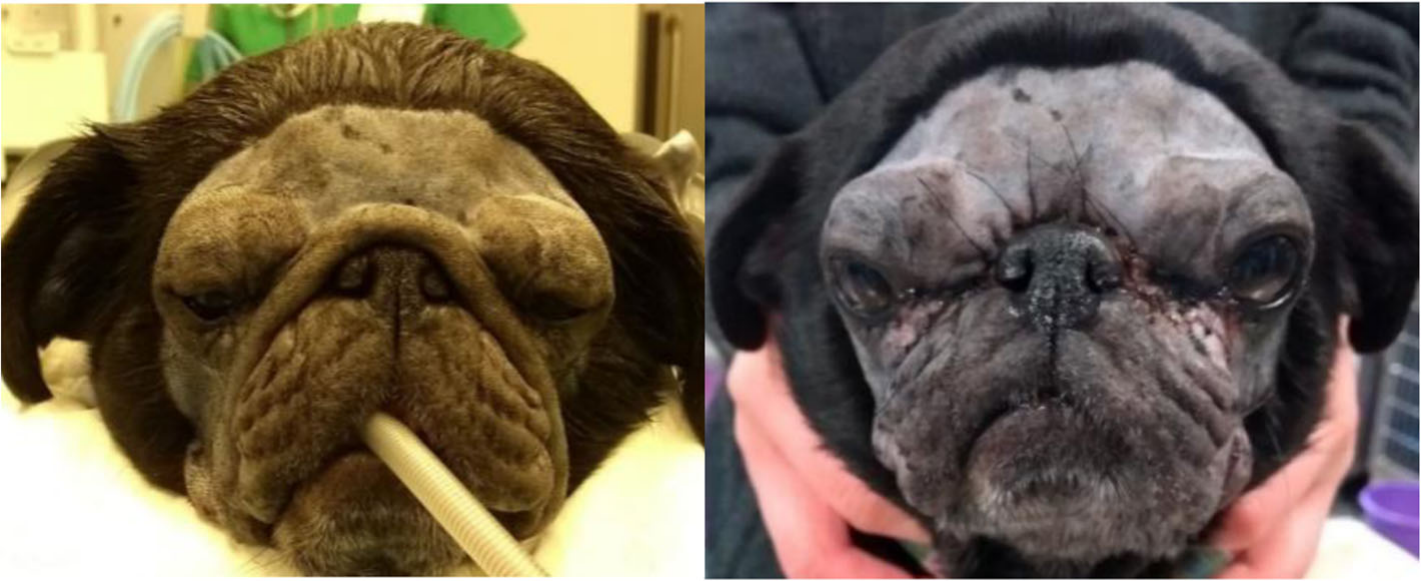
A six-year-old female Pug before and after MCP and resection of the excessive nasal fold. Note that bilateral medial cantoplasty was performed to shorten the eyelid fissure, correcting the lagophthalmia, addressing caruncular trichiasis and correcting the bilateral medial entropion, preserving the lacrimal puncta to avoid epiphora. Moreover, the excessive nasal fold has been trimmed away to prevent facial trichiasis with consequent nasal hair rubbing the cornea leading to pigmentary keratitis formation

Replacement of the gland of the nictitating membrane was achieved in five cases of prolapse (5%, 5/93), through the Morgan and Moore conjunctival pocket technique, and resection of excessive nasal fold was necessary in two Pugs (2%, 2/93) (Fig. 5) [11].

Mass excision was performed in six cases (7%, 6/93), enucleation in three patients (3%, 3/93) and phacoemulsification was helpful in the resolution of two cataract cases (2%, 2/93).

## Discussion

This study described the clinical signs and the medical and surgical management of the brachycephalic ocular syndrome in 93 dogs, allowing for the perception of the incidence of ocular alterations related to the brachycephalic conformation. As these breeds grow in popularity, veterinary hospital teams are treating more and more dogs of brachycephalic breeds with a wide variety of problems caused by breeding for a characteristic short-nosed flat-face.

Some geographical variability was noted during the study period, with Pugs and French Bulldogs apparently more popular in Germany and Portugal respectively.

Some differences between breeds were also noted, yet the low number of individuals from each breed constitutes a major limitation when comparing the same ocular abnormality between a large and a low representative breed.

Concerning ophthalmological examination results, KCS predisposition and primary glaucoma have been described in several brachycephalic breeds. Kitamura et al.demonstrated a difference concerning meibomian glands’ morphology in dogs with normal and low STT values. The most relevant was gland dropout, that was significantly more common in eyes with KCS than in control eyes. Considering that meibomian glands play an important role in the production of the lipid layer of the tear film, its reduced quality is associated with increased evaporation of tear fluid, which likely exacerbates the effects of a quantitative tear deficiency [12].

One English Bulldog with secondary glaucoma due to panuveitis presented with a concomitant ablatio retinae, or retinal detachment [13]. Although infrequent, this condition has been described by Bjerkâs et al. as having a hereditary predisposition in brachycephalic breeds [14].

The fluorescein test was the complementary exam most frequently used and enabled confirmation of 27% of corneal ulcers. The remaining 73% cases were so evident that didn’t need staining to be diagnosed.

Concerning ocular disorders, a mild to severe degree of macroblepahron was frequently seen, which is directly related to the brachycephalic dogs’ skull features, predisposing to corneal dessication, pigmentation and trauma. Together with a varying degree of physiological exophtalmia, macroblepahron can predispose to ocular globe proptosis.

Entropion was the most frequent eyelid-related disorder, diagnosed in 22% of the dogs. Boxer and Pekingese were the only unaffected breeds. This can be attributed to their lower number of representatives or to their conformational specificities. Boxers have longer muzzles with drooling lips, and sometimes are affected with ectropion instead of entropion. Pekingese have thinner noses when compared to French bulldogs, English bulldogs, Shih Tzus and Pugs, and this different nasal conformation can cause less predisposition for medial entropion.

Entropion was always bilateral and medial, plus in one English Bulldog both upper and lower eyelids were affected. This disease was more frequent in Pugs, with 65% of incidence in this breed, followed by English Bulldogs with 60%.

The second most common eyelid-related disorder was distichiasis, with 25% of the cases being bilateral. Trichiasis was important as well, and the majority of animals presented with the caruncular type.

Excessive nasal folds were relatively uncommon and recorded in three Pugs and one Pekinese. Other breeds such as Bulldogs have been linked with some degree of excessive nasal folds [15]. Packer et al. claimed that dogs with excessive nasal folds were five times more likely to develop corneal ulcers, which we could not confirm in this study, since none of the dogs with excessive nasal folds developed corneal lesions [3]. However, the number of dogs diagnosed with that problem is low, which should be taken into consideration.

Corneal ulcers were the second most common alteration in this study. They were present in 44% of the study population and in every breed, except for Boston Terriers and Boxers, with a mean age for this event of 5.65 years. This age does not seem to be in agreement with some of the previous studies, since Moore registered a mean age for this event of 8.2 years, whereas a more recent study by Ramani and co-workers reported a mean age somewhere between 3 months and 3 years [16, 17].

These last authors also reported a higher frequency of bilateral ulcers, rather than the unilateral ones. However, the frequency of unilateral ulcers recorded in the present study was much higher than that for bilateral ones.

It is interesting that only nine cases of ulcers (21% study population) were associated with entropion, ectopic cilia or trichiasis and that the cause of the most corneal ulcers was probably trauma. This supposition is also supported by a study that recorded an incidence of almost 87% ulcerated eyes due to trauma and 13% ulcerated eyes due to a primary cause (ectopic cilia). Nevertheless, the same author did not find a correlation between corneal ulcers and distichiasis or trichiasis [3], whereas we could confirm the existence of such correlation. This last finding is also supported by the studies of Packer et al., because they also described this correlation [3].

Two other corneal alterations were also well represented in our study sample: corneal pigmentary keratitis and fibrosis. Corneal pigmentation affected every breed except for Boston Terriers, and was especially prevalent in Pugs, indicating a possible predisposition to this clinical presentation. Similar findings have been described recently by Maini et al [18] Vallone et al. also reported a greater frequency and severity of corneal pigmentary keratitis in Pugs, in comparison to other brachycephalic breeds [19]. On the other hand, corneal fibrosis was more frequent in the French Bulldog, affecting almost a third of them.

Prolapse of the gland of the nictitating membrane was seen five times and only one animal was greater than 1 year old, which is congruent with the findings of the retrospective study of Mazzuchelli et al., that reported that the prolapse was much more frequent with dogs until 1 year of age [20].

The most frequent lens disorder in our study population was cataracts and it affected three French Bulldogs and two Pugs. Some authors acknowledge an important inheritance component in these breeds for this disease, especially in dogs between 1 and 4 years of age [21, 22]. Three of these dogs were within this age range, while the remaining were older. However, it should be taken in consideration that we present the age at the time of the diagnosis, while the age of onset remains unknown. This means that this disease cannot be attributed with certainty to inheritance or to age consequences.

Some ocular disorders described by Gough and Thomas as being more frequent in the studied breeds such as proptosis, multifocal retinal dysplasia, generalised progressive retinal atrophy and vitreal syneresis were not reported, probably due to the sample size of some breeds included in the study or to the fact that these diseases are not all equally frequent [23].

The most frequent surgical procedure was medial canthoplasty, which is in accordance with other studies [7, 24, 25]. Eleven surgeries were performed, and nine other cases had indication to do so, but had other health problems that required priority in treatment. There was only one registered case of lateral canthoplasty.

Excessive nasal folds were surgically removed in two cases. The third registered case didn’t need this surgical approach because it was submitted to medial canthoplasty, which has itself the potential to straighten the skin below the lower eyelid, and to cover the part of the eye previously exposed to nasal fold trauma, solving different problems at once [25].

Only four out of twenty-three cases of entropion went through a Hotz-Celsus technique, in addition do a medial canthoplasty. In the remaining cases the degree of entropion was not so severe and medial canthoplasty was enough to allow unfolding of the curled medial margin.

All the previously described techniques were successfully applied, and no problems were detected during the dogs’ recovery.

This study described the clinical signs of BOS in a population of brachycephalic dogs, discussed therapeutical approaches and highlighted the importance of responsible breeding, early diagnosis and regular ophthalmic check-ups to correctly diagnose, treat and if possible prevent situations of irreversible blindness in these patients. One can argue that one limitation of this study is the lack of a control group. However, if we consider a group of non-brachycephalic dogs, mesocephalic breeds will not suffer from BOS caused by skull conformation with plane orbits and physiological exophthalmia, macroblepharon, excessive nasal folds and increased corneal exposure, so results on the most common ocular signs caused by these characteristics will not be comparable. In future studies considering a control group of brachycephalic dogs without ocular complaints would be useful, as well as classification of the degree of ocular signs related to BOS syndrome and evaluation of their correlation with the severity of ocular disease.

Another limitation is the sample size of each individual breed, although the aim of this study is not to characterize the predisposition of a BOS breed to a certain ocular presentation. For that purpose, we would need more members of each brachycephalic breed. We hope to adress these issues in future studies.

## Conclusions

The brachycephalic breeds with their big ‘puppy dog eyes’ are popular amongst pet owners. However, they represent a significant animal welfare challenge as they suffer more health problems than breeds with longer snouts.

Brachycephalic Ocular Syndrome is part of the complex Brachycephalic Syndrome, together with the Obstructive Airway Syndrome. Both include different diseases caused by anatomical alterations in these dogs’ oculofacial skull region.

Understanding the morphological modifications in the brachycephalic breeds and being alert to clinical signs is, therefore, essential. Some of the most frequent clinical signs are directly related to the morphological component itself, like entropion and trichiasis. Others are secondarily acquired, including corneal ulcers and corneal dystrophies.

Although the primary, or conformational, abnormalities can’t be adressed, some resulting diseases can be corrected through surgery, improving wellbeing. The rate of success of these techniques is high and the recovery is usually uneventful, leading to a clinical improvement in the short term. When it comes to the secondary developed problems, prognosis is most of the times dependent on how long the primary cause has been present.

Properly informed owners and breeders enable an earlier detection and treatment of the BOS. Moreover, a basic ophthalmological examination should be performed in check-up appointments, particularly in dogs with more extreme features. Some abnormalities are discrete and may pass unnoticed at first sight and many affect dogs at a very young age, which compromises their vision on a long term basis affecting their wellbeing.

This study facilitated the recording of the incidence of ocular alterations related to the brachycephalic conformation. The majority of them presented with medial inferior entropion and caruncular trichiasis. Some differences between breeds were noted, including a higher incidence of corneal pigmentation in Pugs and corneal fibrosis in Shih Tzus, which suggests that some brachycephalic breeds may be predisposed to certain ocular abnormalities. The percentage of secondary problems, like corneal ulcers, was high, which alerts to the importance of a regular ophthalmological check-up, so that an early diagnosis of the primary disorders can be achieved. This will allow for the institution of an adequate treatment, with better prognosis for the patient’s vision and quality of life.

## Data Availability

The datasets used and/or analysed during the current study will be available from the corresponding author on reasonable request.
